# Supplementary figures and images for: Early detection of internet trolls: Introducing an algorithm based on word pairs / single words multiple repetition ratio
Source: PLoS One. 2020 Aug 12;15(8):e0236832. doi: 10.1371/journal.pone.0236832 (PMC7423100; doi:10.1371/journal.pone.0236832)

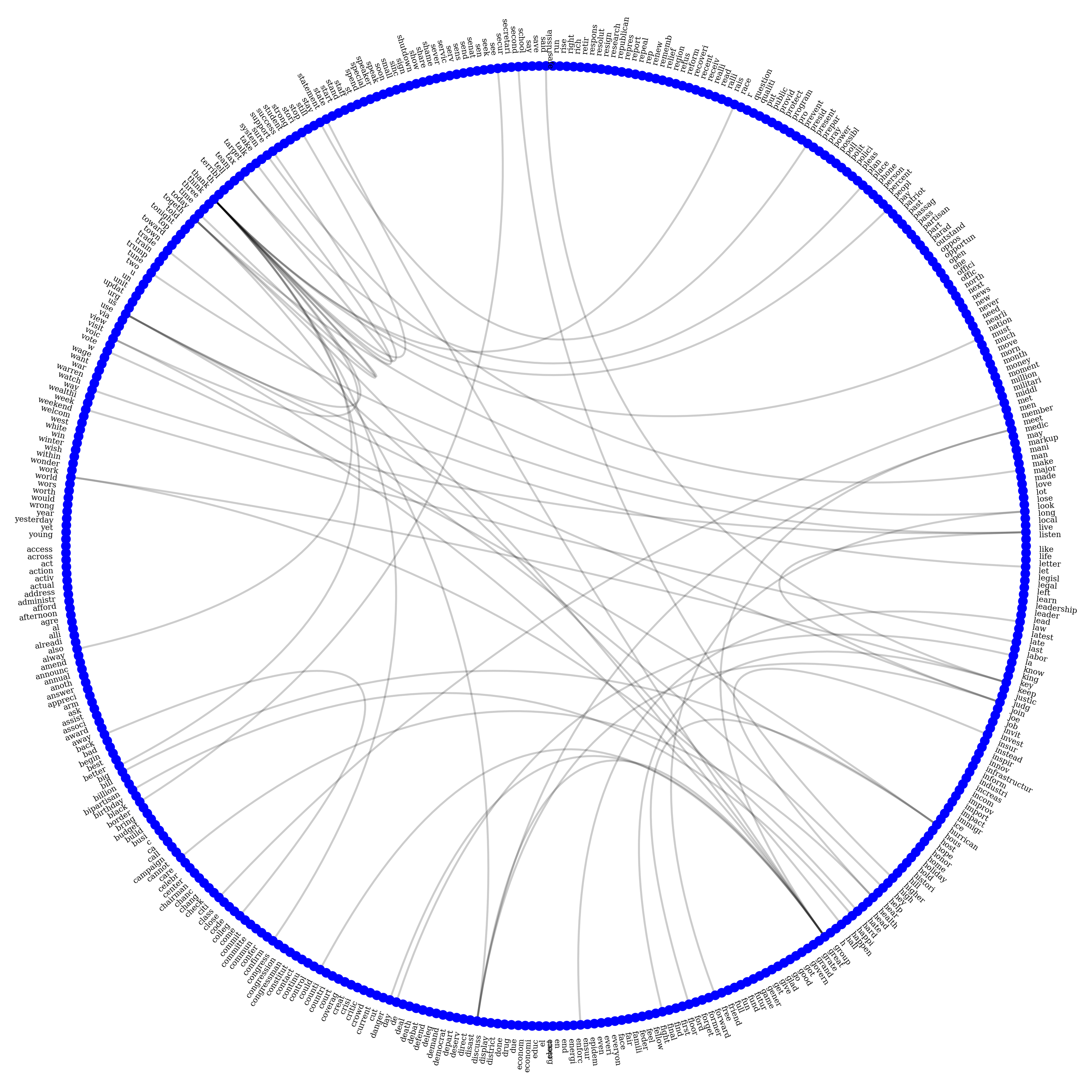

Supplement: S1 Fig — Circos plot of a network of words (nodes) and pairs of words (edges). (TIF) [file pone.0236832.s001.tif]

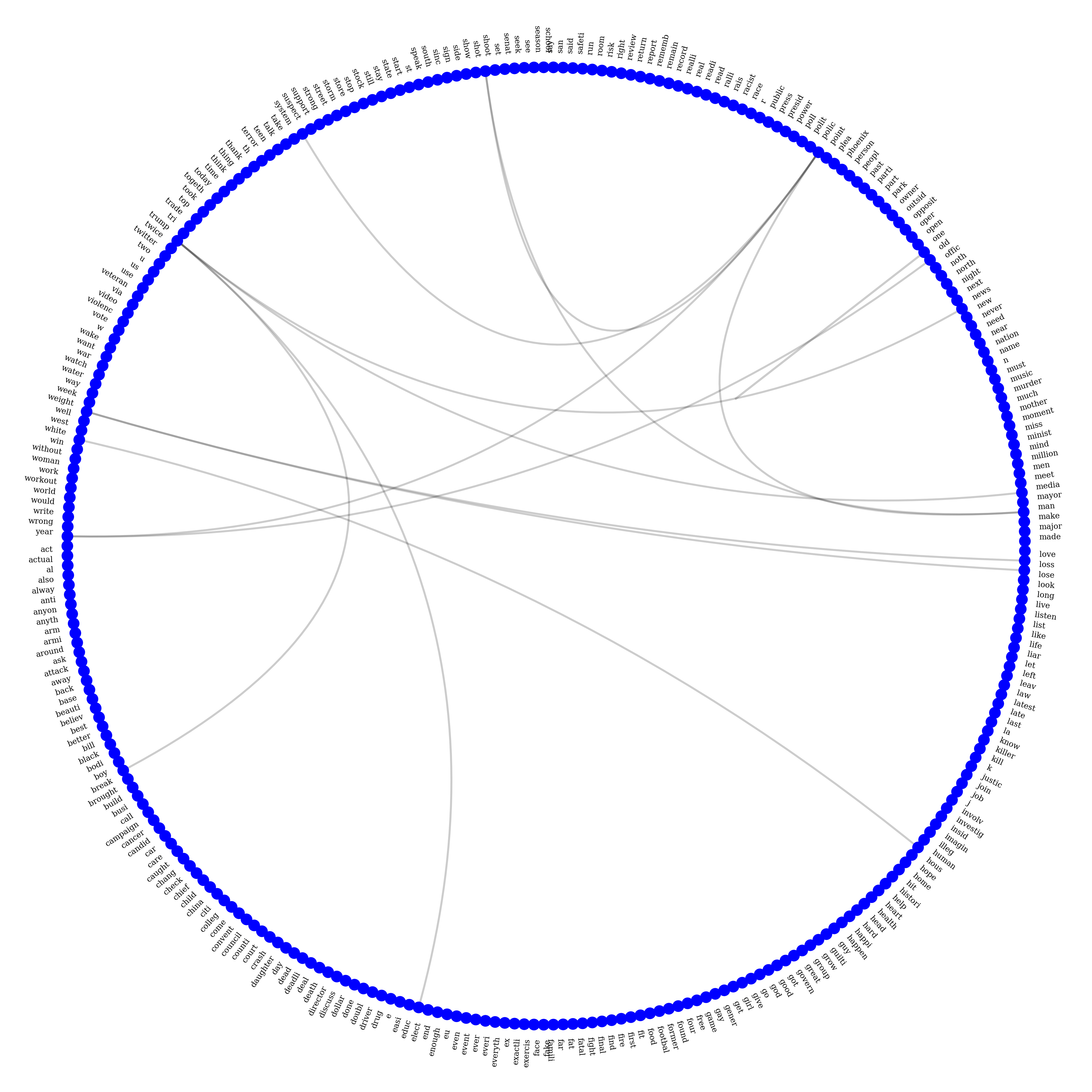

Supplement: S2 Fig — Circos plot of a network of words (nodes) and pairs of words (edges). (TIF) [file pone.0236832.s002.tif]
