# Supplementary material for: Early detection of internet trolls: Introducing an algorithm based on word pairs / single words multiple repetition ratio
Source: PLoS One. 2020 Aug 12;15(8):e0236832. doi: 10.1371/journal.pone.0236832 (PMC7423100; doi:10.1371/journal.pone.0236832)
Supplement: Attachment — Submitted filename: Response to Reviewers.docx [file pone.0236832.s003.docx]

Dear editors and reviewers,

please accept our sincere gratitude for all your comments and suggestions. They helped to improve the paper a lot! Please find below our responses.

| # | Reviewers’ comments | Responses | Lines |
| --- | --- | --- | --- |
| 1 | Please ensure that your manuscript meets PLOS ONE's style requirements, including those for file naming. | Files have been renamed. Figure files have been checked with the Preflight Analysis and Conversion Engine (PACE) digital diagnostic tool. | — |
| 2 | In this case the title is not sufficiently descriptive of the conducted research. | The title has been changed. | 4 |
| 3 | In the Introduction, when the author mentions previous linguistic investigations of trolls’ language, the recent work by Lundberg and Laitinen (2020) might be referenced and briefly discussed. The authors investigated 3.5 million tweets from trolls’ social discourse on Twitter in 2018 but they did not follow a machine learning classification approach as outlined in the current manuscript. Through a simpler frequency analysis, Lundberg and Laitinen also showed that trolls use a smaller linguistic vocabulary, with a sometimes more formal tone. Their results would nicely complement the independent finding presented in this work, on a different dataset and with a different methodology, and could also be interpreted under the light that in order to find more words to express the same concept, not only do trolls mix different contexts but also end up using formal jargon uncommonly used in current language. | Done. | 75-83,  424-427 |
| 4 | In the Introduction, it is not only trolls that represent a powerful weapon for shifting public opinion but also social bots, fake accounts entirely managed by a software. Do social bots fall into the author’s classification of internet trolls? A recent work by Stella et al. (2019) identified a category of “augmented” human Twitter users that achieve trolling by acting normal but then having their messages re-tweeted or favourited by a large number of social bots. | We have good reasons to believe that neither of our datasets contains social bots’ accounts, so we didn’t try to filter out any automatically-generated tweets. | 125-128 |
| 5 | In the Results, Figures 1 and 2 are based on sampling 1000 random tweets. How many of these samplings were performed? | 30 different random samplings were performed. | 134-160 |
| 6 | Figures 3 and 4 seem very difficult to parse. Maybe they could be moved to an Appendix. Also, were these networks based on tweets with the same length? The hierarchical edge bundling visualisation seems to suggest that trolls’ linguistic network is more sparse than non-trolls’ but other than that it is of little use here. | Done. | 181-193,  513-516 |
| 7 | Figure 5-8 seem quite noisy in their left parts. Have they been averaged over 30 different random samplings? As they stand right now, it is really hard to follow the claims from the main text (e.g. two lines “stop mingling after 20 tweets”). Please provide a better labelling, consider changing the scale, smoothing the curves with additional numerical averaging or inserting some insets to highlight specific areas. | Yes, they were averaged over 30 different random samplings. This piece of text has been completely reworked. | 257-281,  302-322 |
| 8 | When reporting the accuracy of the classification, could anything be said about what types of errors does the classification perform, e.g. does it tend to identify “false” trolls or “false” normal users? | Done. | 388-391 |
| 9 | About using Trump’s tweets as a reference corpus for the algorithms, it should be noted that a recent work by Clarke and Grieve (2019) pointed out how Trump’s tweets continuously shift between different linguistic paradigms, which would explain why his language does not have high repetition counts like the ones detected in trolls here. | Done. | 439-441 |
| 10 | Maybe “abusive” is not the correct wording for defining Trump’s tweets in a scientific publication without a supporting reference. It is only rather recently that a third party, in this case Twitter, started analysing the semantic content of Trump’s tweets and identified some of them as “potentially misleading” (CNN, 2020). Maybe the last few sentences of the Discussion might be reworded to accommodate specific references outlining how the POTUS’s tweets are controversial, biased and enflaming. | This passage has been reworded. | 433-436 |
| 11 | Typos: Line 60 – epy widely? | Deleted. | 59 |
| 12 | First, the paper's analysis and results are based on a very small sample of the whole dataset (like 200 or 1000 at some cases tweets out of 1M tweets). This raises concerns on how representative the results are and whether the same results hold if the analysis is done on the whole dataset. I suggest to the authors to expand their analysis so that it includes all the tweets from their dataset rather than sampling a very small percentage of the tweets. | The issue has been addressed. | 392-404 |
| 13 | Second, the authors claim that their approach can be used for other languages as well. I think, since each language its different and each type of state-sponsored trolls might employ different tactics, this is a somewhat unsubstantiated claim. I suggest to the authors to expand their analysis to include data from other languages and types of state-sponsored trolls so that we can better assess the generalizability of the proposed methods. | The issue has been addressed. | 405-415 |
