# Supplementary material for: Early detection of internet trolls: Introducing an algorithm based on word pairs / single words multiple repetition ratio
Source: PLoS One. 2020 Aug 12;15(8):e0236832. doi: 10.1371/journal.pone.0236832 (PMC7423100; doi:10.1371/journal.pone.0236832)
Supplement: Attachment — Submitted filename: Response to Reviewers.docx [file pone.0236832.s004.docx]

Dear editors and reviewers,

please accept our sincere gratitude for your last suggestion. Please find below our response.

| # | Reviewers’ comments | Responses | Lines |
| --- | --- | --- | --- |
| 1 | I am confident that any reader could get the same doubt so the reference to the specific reviewer here could be changed. Also, in order to further dissipate these doubts and the risk of overfitting, I would try to replicate the analysis on the new Table 7 for 100 tweets (which is still a relatively small number) also in case of 250 tweets (which becomes more of a diary/storyline) and, if possible, 1000 tweets. | The reference to the specific reviewer has been changed. The analysis has been replicated for the sample sizes of 100, 250, and 1000 tweets. | 393–404,  412–413 |
